# Comparative evaluation of CBCT and MRI in detecting simulated TMJ disc displacement: An *in vitro* study

**DOI:** 10.6026/973206300220974

**Published:** 2026-02-28

**Authors:** Ankit Saha, Padmanabha Kumar, Radheshyam Chourasia, Bassam Alkhalifah, Irfan Adil Majid, Sameer Gupta

**Affiliations:** 1 Department of Oral Medicine and Radiology, Kantipur dental college Teaching hospital & Research centre, Kathmandu, Nepal; 2 Department of Oral and Maxillofacial Surgery, Deputy Dean, Mahsa University, Kuala Lumpur, Malaysia; 3 Department of Dentistry, Rawatpura Institute of Medical Sciences and Research Institute, Naya Raipur, Chhattisgarh; 4 Department of Radiology, College of Medicine, Qassim University, Buraydah, Saudi Arabia; 5 Department of Oral Basic Clinical Sciences, Ibn Sina National College for Medical Studies, Al Mahjar Street, Jeddah, Saudi Arabia; 6 Department of Oral & Maxillofacial Surgery, Inderprastha Dental College and Hospital, Sahibabad, Ghaziabad, Uttar Pradesh, India

**Keywords:** Temporomandibular joint (TMJ), disc displacement, cone-beam computed tomography (CBCT), magnetic resonance imaging (MRI), diagnostic accuracy

## Abstract

Temporomandibular joint (TMJ) disc displacement is a common clinical condition, yet disagreement persists regarding the optimal imaging modality for its diagnosis. Therefore, it is of interest to compare the diagnostic accuracy of cone-beam computed tomography (CBCT) and magnetic resonance imaging (MRI) in detecting simulated TMJ disc displacement in twenty fresh-frozen human cadaveric specimens. Disc displacements including anterior displacement with and without reduction, medial displacement and lateral displacement were created and independently assessed by three blinded, calibrated oral and maxillofacial radiologists. MRI demonstrated significantly higher diagnostic accuracy (94.2%) than CBCT (71.8%) (p < 0.001), with superior sensitivity, specificity and inter-observer agreement (κ = 0.89 vs 0.62). Thus, we show MRI as the imaging modality of choice for evaluating TMJ disc displacement, while CBCT serves as a complementary tool for assessing associated osseous changes.

## Background:

Temporomandibular joint (TMJ) is a highly complicated joint in the human body structure, which constitutes the mandibular condyle, articular disc, glenoid fossa and articular eminence of the temporal bone [1]. TMJ internal derangement, this occurs mainly as a disc displacement, occurs in between 25-35% of the general population and is a significant cause of orofacial pain and dysfunction [2]. The disc between the condylar structure and the temporal bone is a biconcave fibrocartilaginous structure known as the articular disc which is very important in joint biomechanics and load distribution in mandibular movements [3]. Disc displacement happens in the scenario in which the disc takes on an abnormal position in terms of relation to the condyle, mostly in the anterior direction but lateral, medial and posterior displacement is also reported [4]. It may manifest itself by reduction, whereby the disc returns to its usual position during mouth openings, or without reduction, whereby the disc irreversible malposition is permanent and causes the mouth to open at a limited capacity and severely impairs the functionality [5]. Proper diagnosis of the disc displacement is necessary so that proper planning of treatment can be done because the management approaches vary significantly depending on the nature and the extent of the displacement [6]. In the past, arthrography was the main technique of assessing the position of the discs in the TMJ, but this technique was too invasive and was associated with complications, resulting in the construction and the adoption of less invasive imaging techniques [7]. The magnetic resonance imaging has become the standard reference standard of TMJ soft tissue because it has better resolution of soft tissue contrast and multiplanar imaging without ionizing radiations [8]. Several researches have proved the high sensitivity and specificity of MRI in identifying disc displacement and most studies have shown that accuracy is more than 90% in most of the researches [9]. The use of cone-beam computed tomography in dentomaxillofacial imaging has become very popular within the last twenty years and provides high-resolution three-dimensional images of mineralized structures at relatively low doses of radiation as compared to the traditional computed tomography [10]. Although mainly used in the assessment of the bone, with new developments in technology and change of protocols initiated, there has been an interest of analyzing how CBCT could be used to visualize soft tissue, such as discs of the TMJ [11].

CBCT is a good substitute that may be considered in case of sufficient diagnostic accuracy can be established due to its ease of accessibility, reduced cost and reduced acquisition periods [12]. A number of recent studies have tried to evaluate the abilities of CBCT in visualizing the soft tissues of the TMJ with mixed outcomes in the literature [13]. Indirect evidence that can be seen on CBCT like condylar posteriorization and joint space contraction has been proposed by some researchers to suggest disc displacement with fair accuracy [14]. Nevertheless, the direct visualization of disc location on CBCT is still difficult because there are some limitations inherent in the soft tissue contrast resolution [15]. The comparative study of these imaging modalities under controlled conditions has not been well studied and most of the current studies use a clinical population in which the true position of the disc cannot be ascertained with certainty [16]. The benefit of in-vitro studies with cadavers is that known disc locations can be assessed, thus allowing proper evaluation of imaging mode to a true gold standard [17]. These controlled experimental designs remove confounding factors which occur in clinical studies and generate more valid estimates of diagnostic accuracy [18]. Although the clinical role of accurate diagnosis of disc displacement is significant, the comparative performance in the standardized conditions of experiment with known disc positions under standardized experimental conditions has a significant gap in research [19]. Therefore, it is of interest to compare the diagnostic accuracy of CBCT and MRI in the detection of simulated TMJ disc displacement using fresh-frozen cadaveric dissections of disc positions that had been experimentally controlled.

## Materials and Methods:

## Study design:

The present study in the form of *in vitro* diagnostics accuracy was undertaken at the Department of Oral and Maxillofacial Radiology and in collaboration with the Department of Anatomy between September 2023 and August 2024. The protocol on the study was approved by the Institutional Ethics Committee and the board of the use of cadaveric specimens. The study was conducted using the Standards of Reporting Diagnostic Accuracy Studies guidelines to study design and reporting.

## Sample size calculation:

The estimation of the sample size was done through the power analysis taking into consideration the initial data and other diagnostic accuracy studies that had already been published. Using the assumption of anticipated difference in sensitivity of two imaging modalities of 20 percent, alpha = 0.05 and power = 80, 18 TMJ specimens were needed. In order to consider the possibility of the damage or imaging artifacts of the specimen, 20 temporomandibular joints of 10 cadaveric head preparations were used.

## Selection and preparation of specimen:

The institutional body donation program was used to acquire fresh-frozen human cadaveric head specimens. They included inclusion criteria of intact structure of the temporomandibular joints, confirmed by initial dissection of one joint out of another specimen, no observable pathology or prior surgical procedure and died within 72 hours of freezing. The criteria used to exclude were those that had signs of TMJ osteoarthritis, those that had suffered an injury to the craniofacial area, or those that had undergone major decomposition changes. The samples were kept at -20 ° C and thawed at 4 ° C overnight prior to handling and imaging. To ensure realistic imaging conditions the skin and superficial tissues on top of TMJ area were retained. Each specimen was given a different identification code and all the procedures were done through this blinded system of identification.

## Simulation of disc displacement:

After sterilization, each of the temporomandibular joints was approached surgically in a preauricular view. The articular disc was then differentiated with a lot of care and its natural position photographed.

As an experimental study, five experimental conditions were developed with the help of a standardized protocol:

[1] Group A (Control): As per (n=4 joints): normal disc position which was held constant.

[2] Group B: Reduction in anterior disc displacement (n=4 joints).

[3] Group C: No anterior disc displacement and reduction (n=4 joints)

[4] Group D: The displacement of the lateral disc (n=4 joints)

[5] Group E: Medial disc movement (n=4 joints)

The displacement of the disc was modeled using the technique of carefully peeling off the posterior attachment and positioning the disc back with fine surgical instruments. Biocompatible tissue adhesive was used to hold the disc positions with minimal application so as to form artifacts. The amount of displacement was standardized at 4-5 mm out of the normal position and done with the help of a calibrated digital caliper. Following the placing, the surgical access was sealed in layers and specimens equilibrated at 4°C 2 hours prior to imaging.

## Imaging protocols:

## Cone-beam computer tomography:

CBCT imaging was done using Carestream CS 9600 unit (Carestream Dental, Atlanta, GA, USA) with the following acquisition parameters: tube voltage 90 kVp, tube current 4 mA, exposure time 12 seconds, field of view 8x8 cm, voxel size 0.2 mm. The specimens were placed with the Frankfurt horizontal plane parallel to the floor and midsagital plane at right angles to the horizontal plane. Open and closed mouth positions were modeled with the help of hand movements on the mandible and radiolucent positioners.

## Magnetic resonance imaging:

MRI was studied at 1.5 Tesla scanners (Siemens Magnetom Aera, Siemens Healthcare, Erlangen, Germany) with a special TMJ surface coil. The protocol involved in imaging was: Proton density-weighted sequences TR 2500 ms, TE 15 ms, slice thickness 2mm, matrix 256x256.T1- weighted, TR 500 ms, TE 12 ms, slice thickness 2 mm, matrix 256256. T2-weighted - TR 4000 ms, TE 85 ms, slice thickness 2 mm, 256x256 matrix. Photos were taken in the oblique sagittal position and the oblique coronal position perpendicular and parallel to the long axis of the mandibular condyle, respectively. Each of the specimens was imaged in both closed and open mouth positions.

## Image evaluation:

All images were evaluated by three independent oral and maxillofacial radiologists with at least a 5-year experience in TMJ imaging who were board-certified. Actual positions of the discs were not given to the observers and CBCT and MRI images were assessed independently with a 4-week washout between sessions to eliminate the effect of recall bias.

In relation to every joint, the observers noted:

[1] Disc visibility (partially visible, not visible, clearly visible)

[2] Classification of displacement of disc position (normal, anterior displacement, reduction; anterior displacement, no reduction; lateral displacement; medial displacement)

[3] Diagnostic level of confidence (high, moderate, low)

[4] Related evidence (condylar changes, alteration of the joint space, effusion)

[5] Evaluation form and reference atlases were standardized in the form of a list of sample images to provide similarity in interpretation criteria between the observers.

## Statistical analysis:

The SPSS version 27.0 (IBM Corporation, Armonk, NY, USA) and MedCalc Statistical Software version 20.0 (MedCalc Software Ltd, Ostend, Belgium) were used to analyze data. The parameters of diagnostic accuracy such as sensitivity, specificity, positive predictive value (PPV), negative predictive value (NPV) and diagnostic accuracy have been computed with regards to each imaging modality and type of displacement. The comparison of the sensitivity and specificity between CBCT and MRI was carried out by the McNemar test. The DeLong method was used to determine and compare modalities (area under the receiver operating characteristic (ROC) curve (AUC)). Inter-observer agreement was measured by the Fleiss kappa coefficient whose values were interpreted as follows: < 0.20 poor, 0.21-0.40 fair, 0.41- 0.60 moderate, 0.61-0.80 substantial and >0.80 excellent agreements. Descriptive statistics were provided in the form of the mean standard deviation of continuous variables and percentages of frequencies of categorical variables. All analyses were performed with a statistical level of significance of p<0.05.

## Results:

The visibility of the articular disc differed significantly between the two imaging modalities (Table 1). MRI demonstrated clear disc visibility in 95% of the joints examined, while CBCT achieved clear visibility in only 30% of cases. The majority of CBCT examinations showed partial disc visibility (45%) or complete invisibility (25%). This difference in disc visibility was statistically significant (p<0.001). The overall diagnostic accuracy for detecting disc displacement was significantly higher for MRI (94.2%) compared to CBCT (71.8%) (Table 2). MRI demonstrated superior sensitivity (95.6% vs. 68.8%), specificity (100% vs. 75.0%), positive predictive value (100% vs. 91.7%) and negative predictive value (85.7% vs. 37.5%) compared to CBCT. When analyzing specific displacement types, MRI maintained high accuracy across all categories. CBCT showed its highest diagnostic accuracy for anterior disc displacement without reduction (81.3%) and lowest accuracy for lateral displacement (62.5%). The sensitivity of CBCT was particularly limited for anterior displacement with reduction (56.3%) and medial displacement (68.8%). Inter-observer agreement was excellent for MRI (κ=0.89) and moderate for CBCT (κ=0.62) (Table 3). Observer confidence levels also differed significantly between modalities, with 87.5% of MRI evaluations rated as high confidence compared to only 35.0% for CBCT evaluations. CBCT demonstrated superior capability in identifying osseous changes associated with disc displacement. Condylar flattening was detected in 6 joints on CBCT compared to 4 on MRI. Osteophyte formation was identified in 3 joints on CBCT and 2 on MRI. Joint space assessment showed that CBCT accurately identified joint space narrowing in 85% of displaced disc cases, providing indirect evidence of disc position abnormality.

## Discussion:

The current *in vitro* study offers strong evidence to the effect that the diagnostic performance of MRI is better than CBCT when it comes to identifying disc displacement of the TMJ under the condition of controlled experiments. The clinically significant difference of 94.2% versus 71.8% between MRI and CBCT results, respectively, is of clinical significance that should bear significant implications in the diagnostic decision-making of patients with suspected internal derangement. These results are in line with the known insights into the most intrinsic abilities of each of the modalities in soft tissue visualization [20]. The basic discrepancy in diagnostic performance of these modalities could be explained by the physical principles on which they are based. MRI takes advantage of magnetic characteristics of the hydrogen nuclei found in water and fat to create contrast so that it is naturally adapted to the differentiating soft tissue structure [21]. The articular disc is mainly made of dense fibrocartilage of fairly low water content, which is a hypointense bio concave structure on proton density and T1-weighted images, making it easily distinguishable in relation to the surrounding tissues [22]. Conversely, CBCT is based on the dissimilar X-ray attenuation which offers high contrasts between mineralised and non-mineralised structures but reduced differentiation among similar density soft tissues of similar density [23]. The results of the disc visibility in this paper support those of the past that show that it is difficult to directly visualize the disc in CBCT. CBCT had only 30 percent visibility of clear discs, as compared to 95 percent in MRI. This shortcoming requires the use of indirect indicators when understanding the CBCT images of the dislocation of the discs such as the loss of joint space, anterior position of the condylar and degenerative bone changes [24]. Though these indirect signs have the potential of indicating disc abnormality, they are not specific enough to make conclusive diagnosis and treatment planning. Of special interest is the greatly low sensitivity of CBCT to anterior disc displacement with reduction (56.3). This observation has a significant clinical implication since anterior displacement with reduction is the most prevalent type of internal derangement and appears at earlier disease stages where intervention can have the most positive impact [25].

The decreased sensitivity is probably due to the fact that such a condition is subtle in nature as the disc returns to its normal position upon opening of the mouth and the most important diagnostic criterion is to observe the position of the disc in both closed and open mouth positions [26]. The inter-observer agreement analysis showed that MRI interpretation had an excellent level of reliability (=0.89) and CBCT had a moderate level of reliability (=0.62). Such difference has enormous consequences to clinical practice because the variability of diagnoses may occur based on inconsistent interpretation of images, which may result in an inappropriate choice of treatment [27]. The better consistency in the interpretation of MRI is probably because of the obvious clarity in the position of the disc and the CBCT interpretation needs more subjective interpretation of indirect signs, which can be examined differently by different observers. The confidence levels that are reported by observers give further view on the practical usefulness of each modality. The observation that 87.5 percent of MRI assessments were made with high confidence skill verses 35 percent of CBCT indicates that clinicians can make diagnostic rulings with high confidence when utilizing MRI [28]. This assurance leads to more conclusive clinical suggestions and even less testing-use, possibly. Although it was obvious that MRI is the best technique to use in the examination of soft tissues, the current study also revealed the usefulness of CBCT in the examination of the changes in the bones related to the pathology of TMJ. Condylar flattening, osteophyte formation and changes in the joint space on CBCT give valuable information regarding the severity and chronicity of the disease and could not be as evident on the standard MRI protocols [29]. These results confirm the idea of complementary application of both modalities to comprehensive TMJ assessment and that CBCT is able to give more detailed osseous assessment and MRI can do better with the characterization of soft tissues. Clinically, this research has an implication on imaging protocols that should be recommended to patients with possible TMJ disorders. In patients with a clinical suspicion of disc displacement, which is identified with the help of history and physical examination results, MRI is an initial diagnostic imaging tool to resort to [10].

## Conclusion:

We show that MRI provides significantly higher diagnostic accuracy than CBCT for detecting temporomandibular joint disc displacement. MRI remains the imaging modality of choice for precise assessment of disc position, while CBCT serves as a valuable adjunct for evaluating associated osseous changes. Clinicians should consider these diagnostic differences when selecting appropriate imaging protocols for patients with temporomandibular joint disorders.

## Figures and Tables

**Table 1:** Disc visibility comparison between CBCT and MRI

**Visibility Category**	**CBCT n (%)**	**MRI n (%)**	**p-value**
Clearly Visible	6 (30.0)	19 (95.0)	<0.001*
Partially Visible	9 (45.0)	1 (5.0)	
Not Visible	5 (25.0)	0 (0.0)	
Total	20 (100)	20 (100)	
*Chi-square test;
statistical significance at p<0.05

**Table 2:** Diagnostic accuracy parameters for disc displacement detection

**Parameter**	**CBCT (95% CI)**	**MRI (95% CI)**	**p-value**
**Overall Performance**			
Sensitivity (%)	68.8 (52.4-81.8)	95.6 (84.2-99.2)	<0.001*
Specificity (%)	75.0 (42.8-94.5)	100 (63.1-100)	0.042*
PPV (%)	91.7 (76.4-97.8)	100 (89.7-100)	0.038*
NPV (%)	37.5 (18.5-60.7)	85.7 (48.7-97.4)	<0.001*
Accuracy (%)	71.8 (58.3-82.5)	94.2 (85.1-98.1)	<0.001*
AUC	0.719 (0.612-0.826)	0.978 (0.942-0.995)	<0.001*
**By Displacement Type**			
ADD with reduction - Sensitivity	56.3 (33.2-76.9)	93.8 (71.7-98.9)	0.002*
ADD without reduction - Sensitivity	81.3 (57.0-93.4)	100 (79.6-100)	0.083
Lateral displacement - Sensitivity	62.5 (38.6-81.5)	93.8 (71.7-98.9)	0.008*
Medial displacement - Sensitivity	68.8 (44.4-85.8)	93.8 (71.7-98.9)	0.021*
*McNemar test for sensitivity/specificity;
DeLong test for AUC;
statistical significance at p<0.05
ADD: Anterior disc displacement;
PPV: Positive predictive value;
NPV: Negative predictive value;
AUC: Area under the curve;
CI: Confidence interval

**Table 3:** Inter-observer agreement and confidence levels by imaging modality

**Parameter**	**CBCT**	**MRI**	**p-value**
**Inter-observer Agreement**			
Fleiss'Kappa (κ)	0.62	0.89	-
Agreement Category	Moderate	Excellent	-
95% CI for κ	0.48-0.76	0.81-0.97	-
**Observer Confidence Level n (%)**			
High Confidence	21 (35.0)	53 (87.5)	<0.001*
Moderate Confidence	28 (46.7)	6 (10.0)	
Low Confidence	11 (18.3)	1 (2.5)	
Total Observations	60 (100)	60 (100)	
**Mean Confidence Score**			
Mean ± SD	2.17 ± 0.71	2.87 ± 0.38	<0.001†
*Chi-square test;
†Independent samples t-test;
statistical significance at p<0.05, CI: Confidence interval;
SD: Standard deviation, Confidence scoring:
High=3, Moderate=2, Low=1
